# Percentage signal recovery plus relative cerebral blood volume: a practical dual-parameter strategy for differentiating post-stereotactic radiosurgery tumour progression from radiation necrosis in brain metastases

**DOI:** 10.1186/s13244-026-02240-5

**Published:** 2026-05-21

**Authors:** Vijay Sawlani, Nan Mei, Robert Flintham, Sara Meade, Helen Benghiat, Santhosh Nagaraju, Ismail Ughratdar, Nigel Davies, Ute Pohl, Paul Sanghera, Victoria Wykes

**Affiliations:** 1 University of Birmingham, Birmingham, UK; 2 Department of Imaging, Queen Elizabeth Hospital, University Hospitals Birmingham NHS FT, Birmingham, UK; 3 Department of Radiology, Huashan Hospital, Fudan University, Shanghai, China; 4 Medical Physics, Queen Elizabeth Hospital, University Hospitals Birmingham NHS FT, Birmingham, UK; 5 Oncology, Queen Elizabeth Hospital, University Hospitals Birmingham NHS FT, Birmingham, UK; 6 Pathology, Queen Elizabeth Hospital, University Hospitals Birmingham NHS FT, Birmingham, UK; 7 Neurosurgery, Queen Elizabeth Hospital, University Hospitals Birmingham NHS FT, Birmingham, UK

**Keywords:** Brain metastases, Radiation necrosis, Tumour progression, Dynamic susceptibility contrast, Magnetic resonance imaging

## Abstract

**Objectives:**

Stereotactic radiosurgery (SRS) is widely used for brain metastases, but differentiating tumour progression from radiation necrosis on conventional MRI remains difficult. Percentage signal recovery (PSR), derived from dynamic susceptibility contrast (DSC) perfusion MRI, reflects signal recovery post-contrast and offers insights into capillary permeability. This study aimed to evaluate PSR and relative cerebral blood volume (rCBV) and assess their combined diagnostic value in post-SRS brain metastases.

**Materials and methods:**

Patients with enlarging post-SRS brain metastases and diagnostic uncertainty were retrospectively included. PSR and rCBV were extracted from DSC-MRI and normalised to contralateral white matter. The dataset was split into training and validation cohorts using stratified sampling. Logistic regression with 5-fold cross-validation and bootstrap validation was used. Diagnostic performance was assessed by ROC analysis.

**Results:**

Sixty-one patients (62 lesions; 26 progression, 36 necrosis) were included. Inter-rater reliability was excellent (ICC > 0.90). Progression showed higher rCBV (2.84 vs. 0.76) and lower PSR (95% vs. 176%) (both *p* < 0.001). Both were significant in univariate analysis; PSR remained independently predictive (*p* = 0.04) in multivariate analysis. PSR outperformed rCBV and the combined model in ROC analysis (validation AUCs: 0.960, 0.898, and 0.945, respectively), while the combined PSR and rCBV model maintained excellent sensitivity, specificity, and overall accuracy. Bootstrap-derived thresholds were 108% (PSR) and 1.96 (rCBV). A nomogram was developed for individualised risk estimation.

**Conclusions:**

PSR and rCBV provide complementary diagnostic information for post-SRS lesion assessment. PSR may offer additional value without requiring extra image acquisition, and integration of both parameters could enhance diagnostic confidence. Routine inclusion of PSR and rCBV in post-SRS imaging protocols could be recommended.

**Critical relevance statement:**

This study demonstrates that combining DSC MRI-derived rCBV and PSR improves accuracy and efficiency in distinguishing tumour recurrence from radiation necrosis, offering a practical dual-parameter approach to enhance diagnosis and guide timely clinical decision-making in neurooncology.

**Key Points:**

- DSC MRI-derived rCBV and PSR may aid in improving diagnostic accuracy and reducing time-to-diagnosis in post-SRS brain metastases.
- PSR can be derived from the same DSC acquisition without additional scanning or correction and represents a practical parameter for post-SRS lesion assessment.
- Combining rCBV and PSR may improve diagnostic confidence, especially in equivocal cases, supporting routine use of this dual-parameter model.

**Graphical Abstract:**

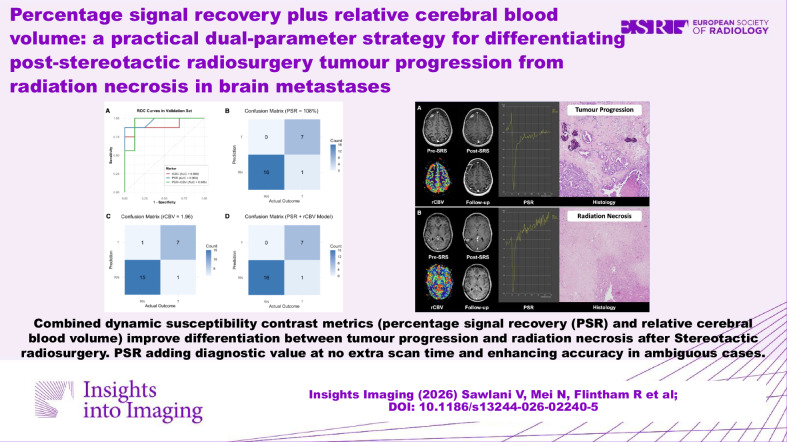

## Introduction

Brain metastases are the most prevalent intracranial tumours in adults, often managed with stereotactic radiosurgery (SRS) or whole-brain radiotherapy. Post-treatment, distinguishing between tumour progression and radiation-induced necrosis remains a significant clinical challenge. Both entities can present similarly on conventional MRI with enlarging contrast-enhancing lesions [1–3]. However, patient management strategies differ significantly, ranging from surgical intervention to continued surveillance or alternative therapies [4].

Various advanced imaging modalities have been investigated to address this diagnostic dilemma, including dynamic susceptibility contrast (DSC) and dynamic contrast-enhanced (DCE) perfusion MRI, magnetic resonance spectroscopy (MRS), treatment response assessment map (TRAM), positron emission tomography (PET), etc. [5–9]. Among these, DSC perfusion-weighted imaging has proven valuable for assessing the microvascular characteristics of brain lesions [10]. Traditionally, relative cerebral blood volume (rCBV) derived from DSC-MRI has been utilised to differentiate recurrent tumours from radiation necrosis, with higher rCBV values typically indicating tumour recurrence due to neovascularisation, and lower values suggesting radiation-induced changes. However, rCBV measurements can be influenced by factors such as contrast agent leakage, blood-brain barrier integrity, and the need for leakage correction, potentially limiting their specificity.

Percentage signal recovery (PSR), another parameter derived from DSC-MRI, reflects the degree of signal intensity recovery after contrast passage and offers insights into capillary permeability and blood-brain barrier integrity [11, 12]. Prior studies have demonstrated that PSR values are typically higher in radiation necrosis compared to tumour recurrence, supporting its diagnostic utility [13]. Despite promising results, PSR has yet to be widely adopted in routine clinical workflows, and its integration alongside rCBV in multivariable diagnostic models remains limited in the literature.

This study aims (1) to assess the diagnostic performance of PSR and rCBV in differentiating tumour progression from radiation necrosis in post-SRS brain metastases, and (2) to evaluate whether combining both parameters improves diagnostic accuracy within a multivariable framework. Through this, we aim to clarify the complementary role of PSR and support its clinical adoption alongside established perfusion markers.

## Materials and methods

This retrospective study was approved by the institution’s review board. Consent has been waived for this retrospective study.

### Study design

Patients diagnosed with brain metastases and treated with SRS between January 2018 to July 2024 were retrospectively retrieved. This study was restricted to cases with brain metastases enlarging post-SRS with uncertainty at the tumour board meeting regarding progression or radiation-related change, impacting the patient’s management.

The inclusion criteria were as follows: (1) diagnosis of brain metastases treated with SRS; (2) at least a 20% volume increase in the largest metastatic lesion on 6-month follow-up MRI after SRS [3]; (3) availability of post-SRS T2*-weighted DSC perfusion imaging and contrast-enhanced T1WI targeting the enlarging lesion. The exclusion criteria were as follows: (1) significant motion artifacts affecting image quality; (2) inability to reconstruct reliable rCBV maps due to technical limitations or poor signal; (3) lack of additional 6-month imaging follow-up, precluding definitive diagnosis of lesion evolution; (4) inability to accurately measure rCBV and PSR due to haemorrhage or marked susceptibility artifacts within the lesions.

The diagnosis was confirmed through clinico-radiological evaluation, with at least a 6-month follow-up in the majority of cases, and histological confirmation was available in those who underwent surgical resection during follow-up. For some lesions, surgical resection was performed after SRS during follow-up, either for diagnostic confirmation or further therapeutic purposes.

### MR imaging

T2* DSC perfusion imaging was performed on a 3-T scanner (Magnetom Skyra; Siemens Healthineers) with a 32-channel phased-array head coil using gradient-echo-EPI during the first pass of a standard dose (7.5 mmol) bolus of gadolinium-based contrast agent (Gadovist, Bayer Schering Pharma) administered intravenously at a flow rate of 6 mL/s.

Eighty imaging volumes were acquired at a temporal resolution of 2.1 s with the bolus typically arriving between the 10th and 15th volume. PWI acquisition parameters were TR/TE = 2100/30 ms, flip angle = 60°, and spatial resolution = 1.5 mm (in-plane) × 4 mm (slice thickness). This was followed by a post-contrast 3D T1WI MPRAGE sequence acquired in the axial plane with sagittal and coronal reformats.

### rCBV analysis

DSC perfusion data were post-processed using the SyngoVia platform (MR Neurology workflow, software version VB80; Siemens) using a global arterial input function and without leakage correction, and rCBV maps were generated. For each lesion, three circular regions of interest (ROIs) with a diameter of 3 mm were manually placed within the enhancing component by two independent neuroradiologists, each with 25 and 5 years of working experience, who were blinded to the clinical outcome and pathology. Utmost care was taken to avoid the region of haemorrhage and vessels in post-SRS lesions, which can distort the accuracy of rCBV measurements due to susceptibility artefacts and signal contamination. The maximum mean value among three ROIs of equal size placed within the enhancing portion of the lesion was selected, aiming to represent the most biologically active or aggressive region of the tumour, in line with prior studies differentiating tumour recurrence from treatment-related effects [14]. To standardise measurements across patients, this value was normalised to the mean rCBV obtained from a mirror ROI placed in the contralateral normal-appearing white matter on the same slice and at the same anatomical level as the lesion, preferentially within the centrum semiovale, while carefully avoiding areas of leukoencephalopathy or other signal abnormalities. The resulting ratio was defined as the rCBV ratio. To assess reproducibility, the intraclass correlation coefficient (ICC) was calculated for rCBV [15].

### PSR quantification

PSR values were calculated from the same three ROIs used for rCBV measurement, and the mean of these three ROIs was used for each lesion, followed by normalisation to contralateral normal-appearing white matter to reduce inter-subject variability. The T2*-weighted signal intensity-time curves within each ROI were extracted, and PSR was calculated using the formula [11]:

$${{\rm{PSR}}} = 100 \% \, \times [({S}_{{\rm{post}}}-{S}_{{\min}})/({S}_{{\rm{pre}}}-{S}_{{\min}})] ,$$

where ${S}$_pre_ is the mean signal intensity of the first five pre-bolus baseline points before contrast arrival, ${S}$_min_ is the minimum signal during the first pass of the contrast bolus, and ${S}$_post_ is the mean signal intensity of the last five points after contrast passage. To assess reproducibility, ICC was also calculated for PSR.

### Statistical analysis

Normality of continuous variables was assessed using the Shapiro–Wilk test. Continuous variables were compared using either independent *t*-tests or the Wilcoxon rank-sum tests, depending on distribution. Categorical variables were analysed using Chi-squared or Fisher’s exact tests, as appropriate. Data distributions of PSR and rCBV were visualised using violin plots combined with box plots.

To ensure model robustness and minimise overfitting, the dataset was randomly divided into a training cohort (60%) and an independent validation cohort (40%) using stratified sampling based on follow-up outcome. Within the training set, univariate and multivariate logistic regression analyses were conducted to assess the predictive value of PSR and rCBV in post-SRS tumour assessment. Model development incorporated 5-fold cross-validation for variable selection and hyperparameter tuning. Receiver operating characteristic (ROC) analyses were conducted for PSR, rCBV, and the combined model, with optimal cut-offs determined by maximising the Youden index through bootstrap resampling. The best-performing model was then applied to the validation set. To evaluate the model’s generalisability, bootstrap resampling was performed on the testing set to validate predictive accuracy metrics, including the area under the curve (AUC), sensitivity, specificity, and accuracy. Pairwise AUC comparisons were performed using DeLong’s test to assess incremental diagnostic value. A nomogram was constructed from the final multivariable model to enable individualised clinical risk estimation.

Statistical significance was defined as *p* < 0.05. All analyses were performed using R software (version 2024.12.0) [16].

## Results

### Patient cohort

A total of 61 patients met the final inclusion and exclusion criteria. As one patient presented with two lesions showing comparable enlargement of more than 20%, a total of 62 lesions were included in the final analysis. Among these, 26 were histologically or clinically diagnosed as tumour progression and 36 as radiation necrosis. Baseline characteristics of both groups are detailed in Table 1.

**Table 1:** Baseline clinical and imaging characteristics between the tumour progression and radiation necrosis groups

	Tumour progression (*n* = 26)	Radiation necrosis (*n* = 36)	*p*-value
Age in years (mean ± SD)	57 ± 9	63 ± 10	0.03^a*^
Sex, male:female	4:22	12:24	0.14^b^
Primary tumour (count)			0.03^b*^
Breast	18	10	
Lung	3	9	
Melanoma	3	8	
Gynaecology	1	2	
Neuroendocrine	0	1	
Rectal	1	2	
Renal	0	4	
Non-brain metastasis, yes:no	5:21	3:33	0.26^b^
Surgery on brain metastasis during follow-up, yes:no	17:9	12:24	0.02^c*^
Chemotherapy, yes:no	19:7	18:18	0.11^c^
Targeted therapy or immunotherapy, yes:no	19:7	19:17	0.17^c^
Follow-up, alive:dead	7:19	22:14	0.01^c*^
rCBV (mean ± SD)	2.84 ± 1.21	0.76 ± 0.44	<0.001^d*^
PSR (mean ± SD)	95% ± 19%	176% ± 48%	<0.001^d*^

* *p*-value < 0.05

^a^ *t*-test

^b^ Fisher’s exact test

^c^ Chi-squared test

^d^ Wilcoxon rank-sum test

No significant differences were observed between the tumour progression and radiation necrosis groups in terms of sex distribution, primary tumour type, or receipt of systemic therapy (all *p* > 0.05). However, patients with radiation necrosis were significantly older (63 ± 10 years vs. 57 ± 9 years, *p* = 0.03) and more likely to be alive at follow-up (*p* = 0.03). Surgical resection with histopathological confirmation was more common in the tumour progression group (17/26) than in the radiation necrosis group (12/36) (*p* = 0.02), with the remaining cases diagnosed based on follow-up imaging. The distribution of primary tumour sites also differed significantly between groups (*p* = 0.03), with breast cancer being the most common in both groups (tumour progression: 18; radiation necrosis: 10), followed by lung and melanoma metastases.

**Fig. 1:**
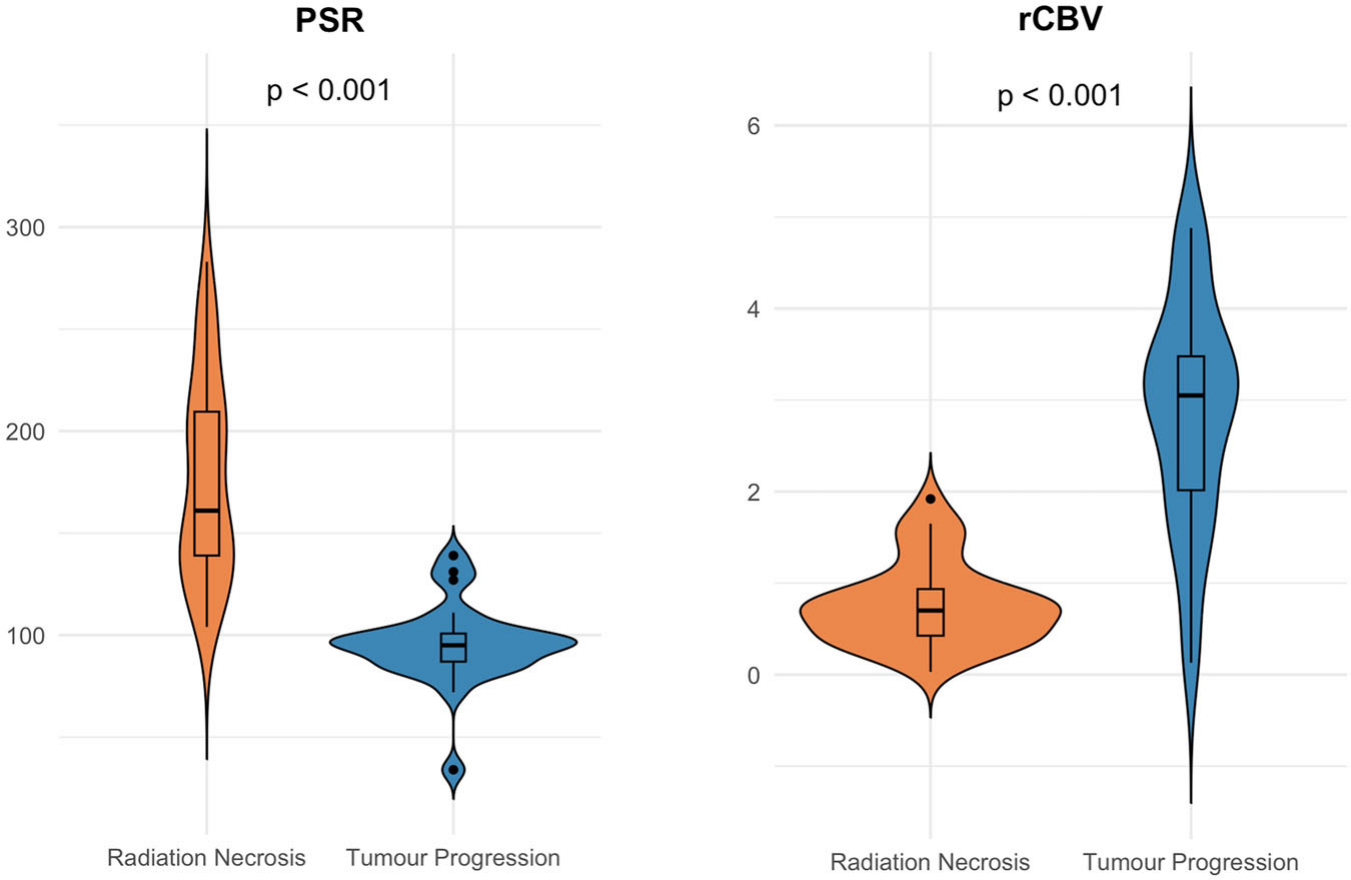
Violin and box plots show the distribution of PSR and rCBV in tumour progression and radiation necrosis groups, including median, interquartile range and *p*-value

ICCs exceeded 0.90 for both rCBV and PSR, indicating excellent inter-rater reliability. Significant differences in perfusion parameters were observed between the two groups. Mean rCBV was markedly higher in the tumour progression group compared to the radiation necrosis group (2.84 vs. 0.76, *p* < 0.001), whereas mean PSR was significantly lower (95% vs. 176%, *p* < 0.001) (Fig. 1).

The entire dataset was randomly divided into a training cohort (*n* = 38, 60%) and an independent validation cohort (*n* = 24, 40%) using stratified sampling based on follow-up outcome to ensure balanced groups. There were no significant differences between the two cohorts in terms of demographic characteristics, primary tumour types, presence of non-brain metastases, treatment modalities, or survival status (Supplementary Table 1). Quantitative imaging parameters, including rCBV and PSR, were also comparable between the training and validation sets (*p* = 0.76 and *p* = 0.75, respectively).

### Logistic regression analysis

Univariate logistic regression analysis performed in the training cohort demonstrated that both PSR and rCBV were significantly associated with differentiation between tumour progression and radiation necrosis (PSR: odds ratios (OR) = 0.91, 95% CI: 0.84–0.95, *p* < 0.001; rCBV: OR = 8.96, 95% CI: 2.93–50.59, *p* < 0.001; Table 2). Specifically, lower PSR values were significantly associated with tumour progression, indicating that higher PSR is more likely related to radiation necrosis. Conversely, higher rCBV values were associated with an increased likelihood of tumour progression.

**Table 2:** Univariate and multivariate logistic regression analysis for differentiating tumour progression from radiation necrosis using PSR and rCBV in the training set

	Univariate		Multivariate	
	PSR	rCBV	PSR	rCBV
Estimate	−0.09	2.19	−0.06	0.98
OR (95% CI)	0.91 (0.84–0.95)	8.96 (2.93–50.59)	0.94 (0.89–0.99)	2.66 (0.72–9.82)
*p*-value	< 0.001*	< 0.001*	0.04*	0.14
AIC^#^	26.37	33.26	25.59	

*OR* odds ratio, *AIC* Akaike Information Criterion

* *p*-value < 0.05

^#^ Lower AIC value indicates better model fit, penalising for model complexity

**Fig. 2:**
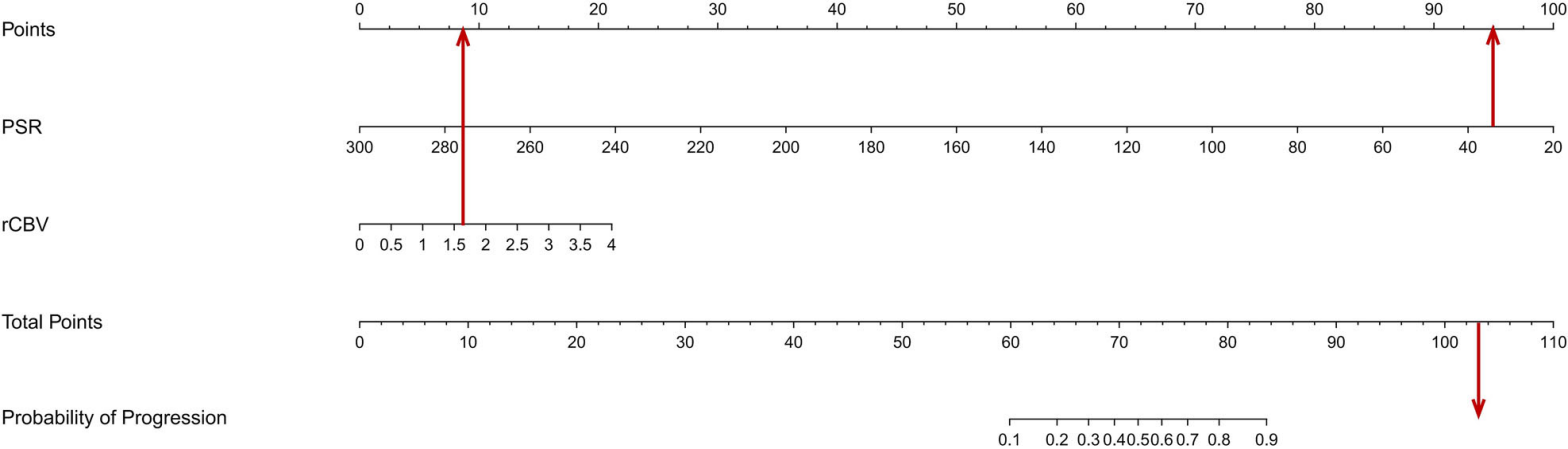
Nomogram integrating rCBV and PSR for the prediction of post-SRS tumour progression. Each parameter is mapped to a corresponding point value on the top axis. The total score, calculated by summing individual points, aligns with the estimated risk of progression indicated on the bottom axis. In a representative case, Patient_38: a 57-year-old female with a history of breast cancer who was deceased at follow-up, demonstrated an rCBV of 1.59 and a PSR of 34%. Despite the borderline rCBV, the markedly reduced PSR contributed to a high total score, consistent with a high predicted probability of progression and supporting the diagnosis of tumour recurrence

In multivariate analysis, PSR remained an independent and statistically significant predictor (OR = 0.94, *p* = 0.04), while the association of rCBV with tumour progression did not reach statistical significance (OR = 2.66, *p* = 0.14). These findings suggest that PSR provides additional and more robust diagnostic value beyond rCBV in distinguishing tumour progression from treatment-related changes. Variance inflation factors for both variables were below 5, indicating minimal multicollinearity. A nomogram was developed based on the multivariable logistic regression model incorporating both rCBV and PSR to estimate the probability of tumour progression following SRS, providing an intuitive and quantitative method for integrating perfusion metrics into individualised clinical decision-making (Fig. 2).

### Diagnostic performance assessment

**Fig. 3:**
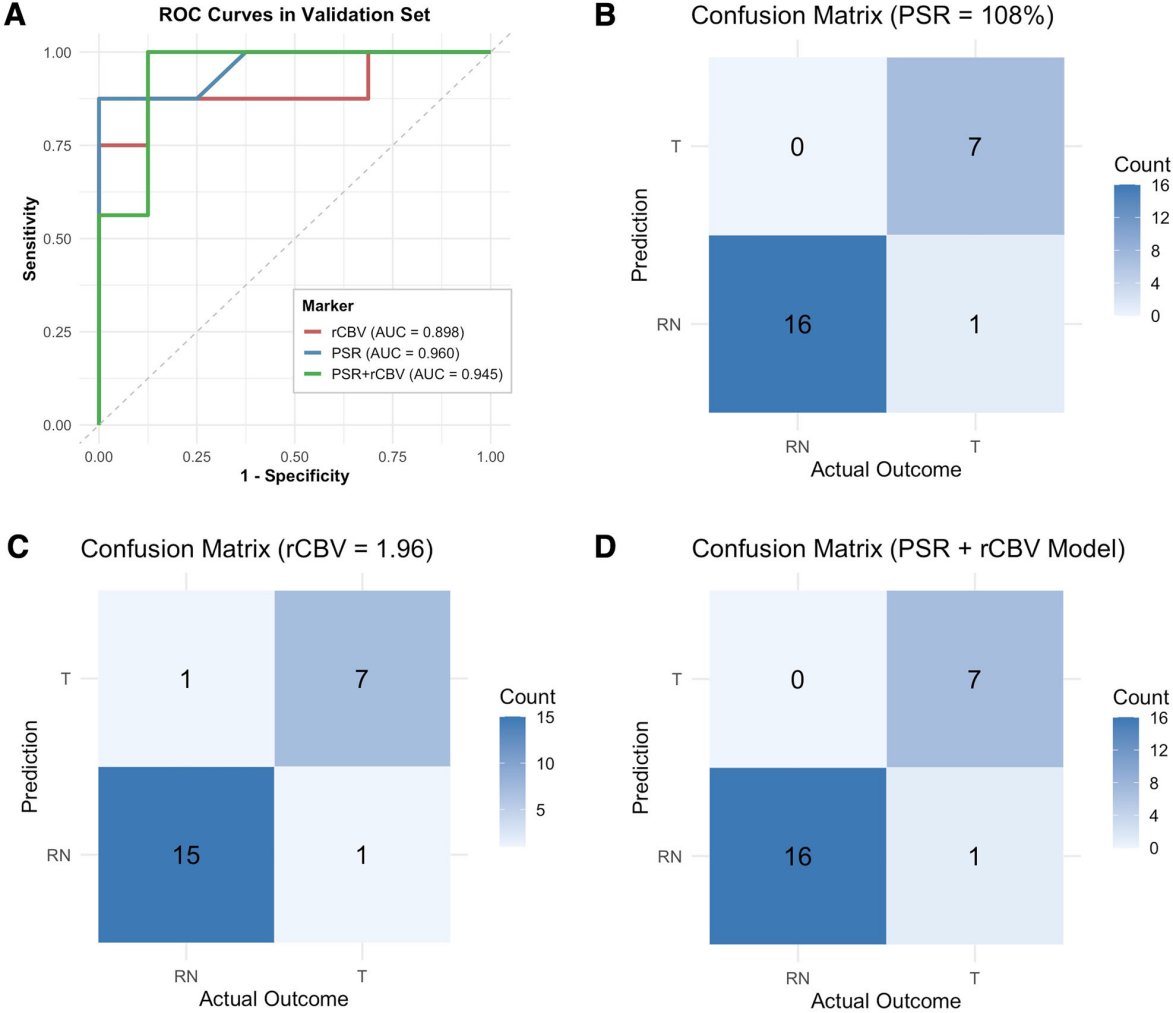
Comparative ROC curves and confusion matrices of PSR, rCBV, and the combined model for differentiating tumour progression from radiation necrosis in the validation set. **A** ROC curves. **B**–**D** Confusion matrices of the PSR, rCBV, and combined models, respectively

ROC curve analysis illustrated that PSR alone provided excellent diagnostic accuracy for differentiating tumour progression from radiation necrosis, with a mean AUC of 0.987 ± 0.02 in the training set and a bootstrap-corrected AUC of 0.960 (95% CI: 0.812–1.000) in the validation set (Table 3, Fig. 3). The corresponding accuracy, sensitivity, and specificity in the validation cohort were 95.8%, 87.5%, and 100%, respectively. The rCBV alone model demonstrated robust performance, with a mean training AUC of 0.950 ± 0.11 and a validation AUC of 0.898 (95% CI: 0.695–1.000), alongside an accuracy of 91.7%, sensitivity of 87.5%, and specificity of 93.8%. Optimal cut-off values for PSR and rCBV, derived by maximising the Youden index through bootstrap resampling, were 108% and 1.96, respectively. The combined PSR and rCBV model demonstrated comparable diagnostic performance, with a mean training AUC of 0.987 ± 0.02 and a validation AUC of 0.945 (95% CI: 0.859–1.000). In the validation cohort, the accuracy, sensitivity, and specificity were 95.8%, 87.5%, and 100%, respectively, matching the performance of the PSR-alone model.

**Table 3:** Diagnostic performance of PSR, rCBV, and the combined model for differentiating tumour progression from radiation necrosis in the training set and validation set

	Training set Radiation necrosis (*n* = 20) Tumour progression (*n* = 18)	Validation set Radiation necrosis (*n* = 16) Tumour progression (*n* = 8)			
Model	AUC (mean ± SD)	AUC (Bootstrapped 95% CI)	Accuracy	Sensitivity	Specificity
PSR alone (cut-off = 108%)	0.987 ± 0.02	0.960 (0.812–1.000)	95.8%	87.5%	100%
rCBV alone (cut-off = 1.96)	0.950 ± 0.11	0.898 (0.695–1.000)	91.7%	87.5%	93.8%
PSR + rCBV	0.987 ± 0.02	0.945 (0.859–1.000)	95.8%	87.5%	100%

## Discussion

The limited predictive value of conventional contrast-enhanced MRI in assessing treatment response after SRS for brain metastases has driven the exploration of advanced imaging techniques. Methods such as perfusion MRI, MRS, TRAM, and PET have been investigated to address the diagnostic dilemma. A recent meta-analysis by Teunissen et al reported pooled sensitivities and specificities for individual modalities as follows: anatomical MRI (79% sensitivity, 76% specificity), DCE (74%, 92%), DSC (83%, 78%), and MRS (80%, 78%) [17].

While DCE and MRS provide valuable physiological and metabolic information, their clinical utility is often constrained by limited spatial resolution, susceptibility to motion artefacts, prolonged acquisition times, and the need for specialised post-processing. TRAM imaging requires complex image registration and is not widely accessible, and PET has yet to demonstrate consistent superiority over perfusion MRI in this context [18–21]. Importantly, the meta-analysis highlighted that no single imaging modality could offer a universally reliable diagnosis, with performance influenced by institutional expertise and clinical context. Multiparametric approaches that combine complementary imaging techniques have shown the highest diagnostic accuracy, underscoring the benefit of integrated imaging biomarkers for improved differentiation between tumour progression and radiation necrosis.

Given the limitations of multiple advanced techniques, there is a clear need for practical, accessible, and easily applicable imaging biomarkers that can be integrated into routine post-SRS brain metastases follow-up. Tumour recurrence is associated with increased angiogenesis and hyperperfusion, characterised by higher blood volume, while radiation-induced changes show hypoperfusion due to endothelial damage and necrosis [22, 23]. DSC-MRI remains a clinically feasible and validated method for treatment response assessment. Within its perfusion-derived haemodynamic parameters, rCBV serves as a marker of tumour angiogenesis, microvascular proliferation. Several studies have proposed rCBV cut-off ratios for tumour recurrence, such as > 1.74 (sensitivity 96.3%, specificity 86.2%), > 2.0 (sensitivity 85%, specificity 71.4%) and > 2.1 (sensitivity 100%, specificity 95.2%) [5–7].

PSR, another DSC-derived parameter, reflects capillary permeability and contrast leakage dynamics [24]. Importantly, it is calculated from the same DSC acquisition as rCBV, without requiring dual-bolus protocols or leakage correction. Compared to rCBV, PSR is less susceptible to susceptibility artefacts and modelling assumptions, making it more practical for clinical use. Prior studies have shown PSR’s utility in differentiating glioma recurrence from radiation necrosis, assessing post-SRS response in brain metastases, aiding CNS lymphoma diagnosis, and contributing to imaging-based differential diagnosis of brain tumours [11, 13, 24–28]. Despite its potential, PSR remains less adopted in clinical practice for differentiating post-SRS tumour progression from radiation necrosis.

**Fig. 4:**
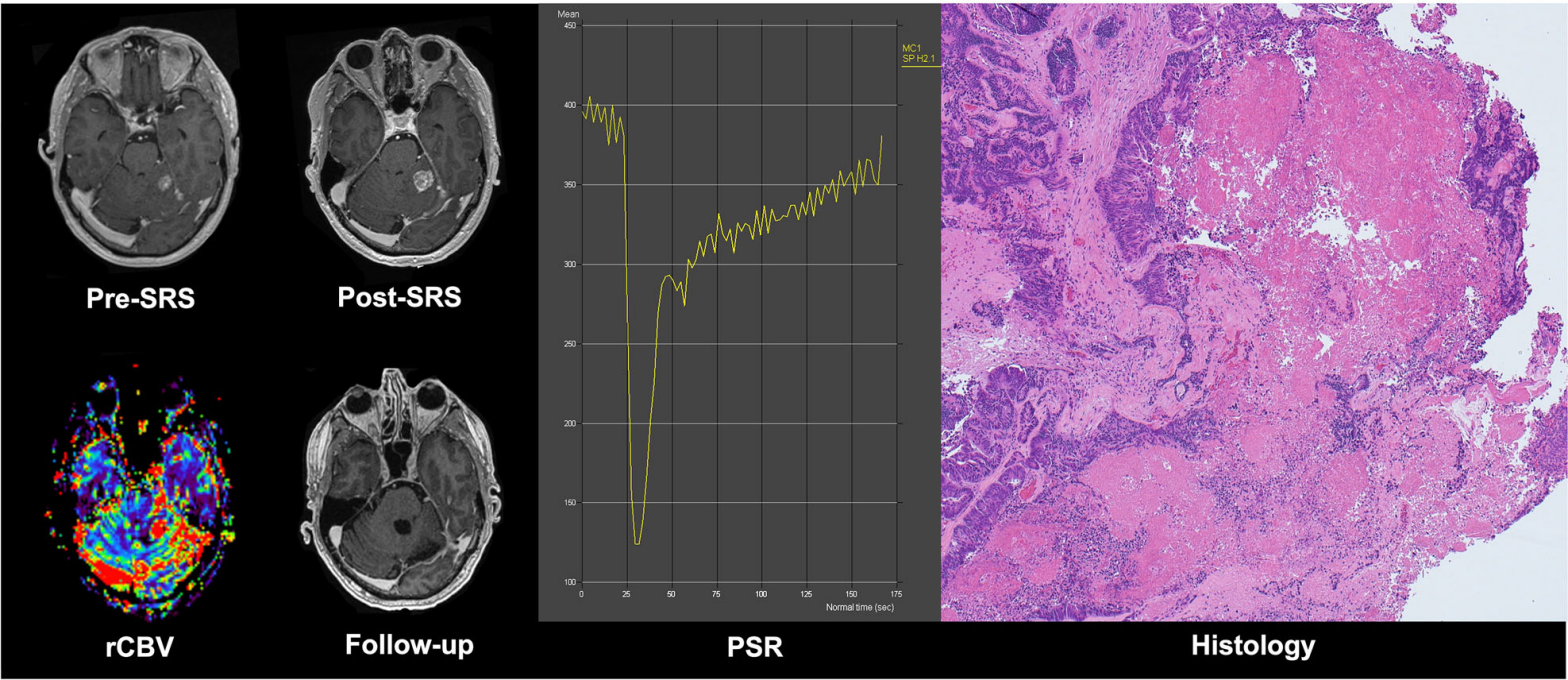
Representative case of tumour progression following post-SRS evaluation in a brain metastasis patient. Patient_14: A 63-year-old male with a history of rectal cancer who was deceased at follow-up. Pre-SRS T1CE shows a solitary metastasis in the left cerebellar hemisphere. Post-SRS 6-month follow-up T1CE image demonstrates enlargement of the lesion. Post-SRS rCBV map shows a high rCBV value of 5.8, and the representative DSC-derived signal intensity-time curve shows a low PSR of 87%. Follow-up T1CE image 3 months later was followed by surgical resection. Histology has confirmed viable metastatic adenocarcinoma with an intestinal pattern on the left, with patchy intertumoural necrosis and haemorrhage on the right on hematoxylin and eosin (H&E) staining at × 40 magnification

**Fig. 5:**
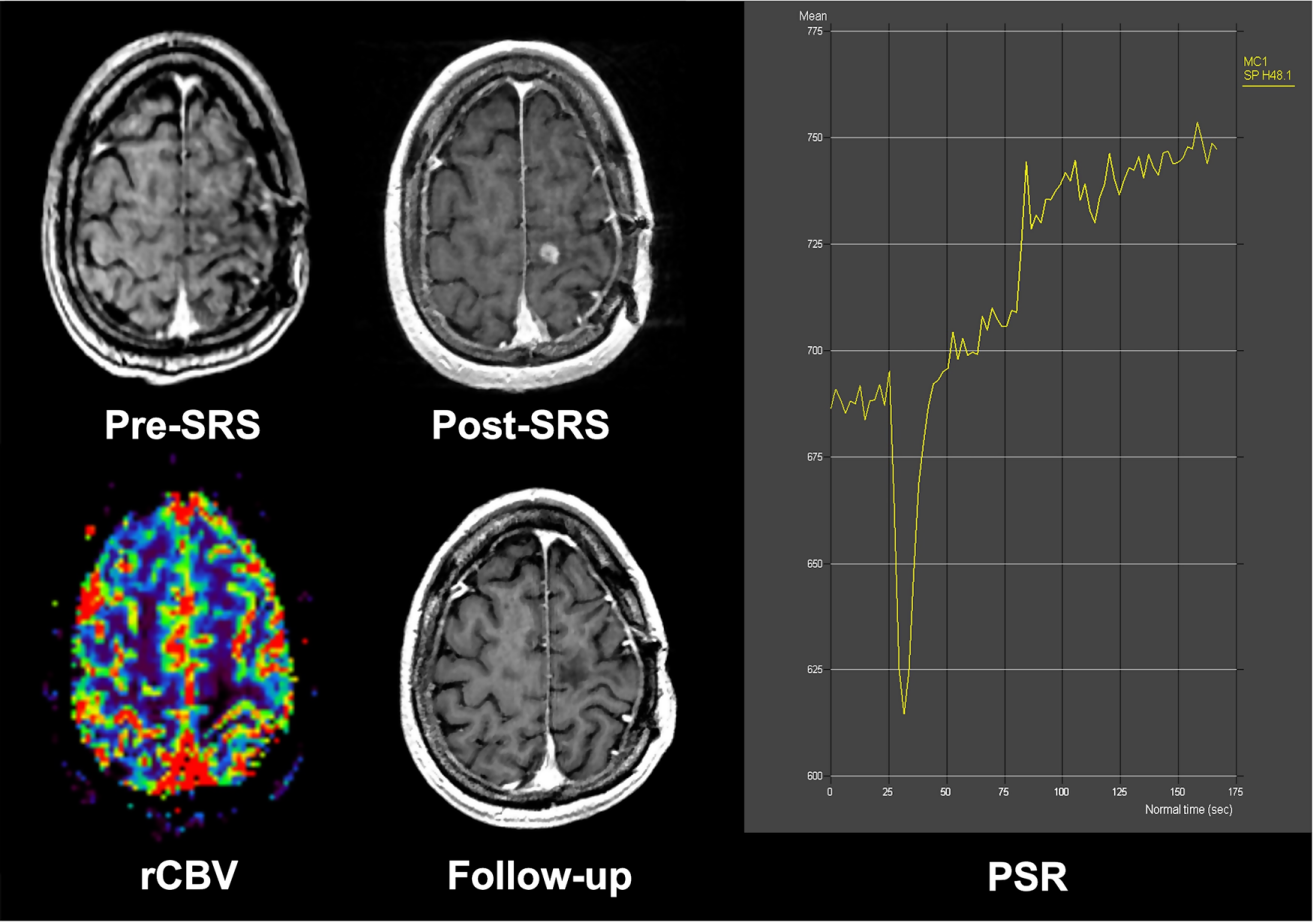
Representative case of radiation necrosis following post-SRS evaluation in a brain metastasis patient. Patient_15: An 80-year-old female with a history of lung cancer, currently alive at follow-up. Pre-SRS T1CE shows a solitary metastasis in the left posterior frontal lobe. Post-SRS T1CE at 6 months demonstrates lesion enlargement. rCBV map at 6-month follow-up shows a low rCBV value of 0.34, and a corresponding DSC-derived signal intensity-time curve shows a high PSR of 182%. Follow-up T1CE at 12 months shows lesion resolution, consistent with radiation necrosis change

The biological mechanism underlying treatment effects after SRS is not fully understood but is thought to involve radiation-induced microvascular injury, leading to indirect tumour cell death [29]. The subsequent release of intracellular toxins may contribute to vasogenic oedema, inflammation, and disruption of blood-brain barrier integrity, all of which can affect perfusion and permeability characteristics on DSC imaging [30]. By combining rCBV and PSR, respectively representing perfusion and permeability, complementary physiological information can be obtained. This integrated approach may enhance diagnostic confidence and improve the accuracy of post-treatment response assessment in brain metastases. Our findings support this hypothesis, demonstrating that PSR provides additive diagnostic value beyond rCBV in distinguishing tumour progression (Fig. 4) from radiation necrosis (Fig. 5).

In our study, patients with radiation necrosis were significantly older and more likely to be alive at follow-up, reflecting a more indolent clinical trajectory. Both rCBV values above 1.96 and PSR values below 108% were strongly associated with tumour progression following SRS. PSR alone demonstrated superior diagnostic performance compared to rCBV, achieving higher AUC values (0.960 vs. 0.898 in the validation cohort). These findings align with prior studies validating the role of rCBV in identifying recurrent disease [17, 31, 32] and further support previous reports indicating the potential diagnostic utility of PSR [13, 24].

The combined PSR and rCBV model demonstrated excellent diagnostic performance in the validation cohort, with an accuracy of 95.8%, sensitivity of 87.5%, and specificity of 100%. The model’s AUC was 0.945 (95% CI: 0.859–1.000), reflecting robust discrimination between tumour progression and radiation necrosis. Although the PSR-alone model showed a slightly higher AUC of 0.960, this difference was not statistically significant, and its performance was less stable with a wider confidence interval (0.812–1.000). It suggested that PSR and rCBV might provide complementary but partially overlapping physiological information, which stems from their shared dependence on perfusion dynamics or limitations related to sample size. Nevertheless, PSR demonstrated clear added value in clinical practice, particularly in cases with borderline rCBV values. For example, in one representative case, an rCBV of 1.59 alone was equivocal; however, a markedly low PSR of 34%, along with a high nomogram-predicted risk, supported a diagnosis of tumour progression, later confirmed by histology (Fig. 2, Supplementary Fig. 1). Such cases underscore the potential of PSR to enhance diagnostic confidence and serve as a useful adjunct to rCBV, especially when used within a multivariable framework.

**Fig. 6:**
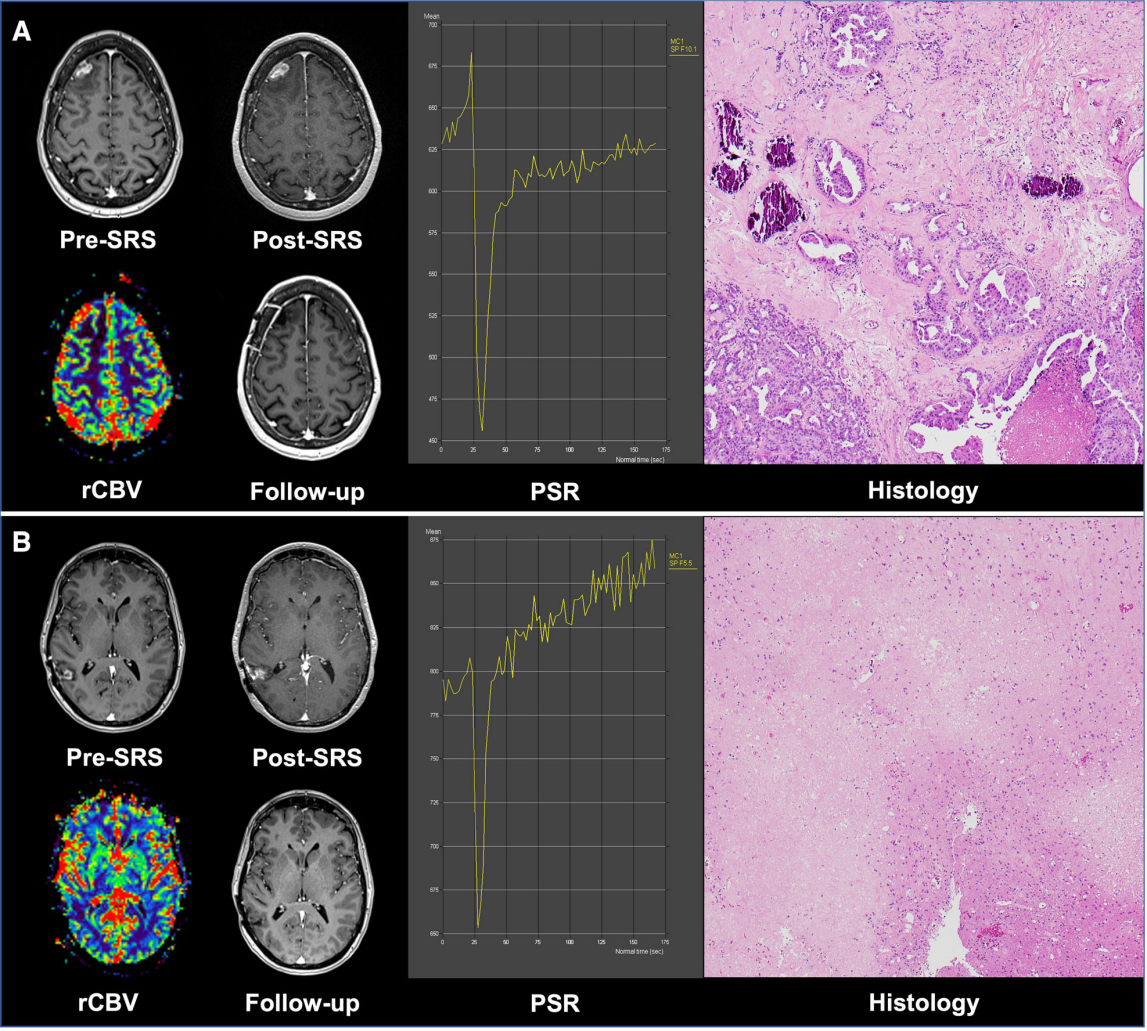
Representative case demonstrating both tumour progression and radiation necrosis in two distinct lesions in a patient with brain metastases following SRS. Patient_37: A 56-year-old female with a history of breast cancer who was deceased at follow-up. **A** Right frontal lobe metastasis lesion showing tumour recurrence: pre-SRS T1CE shows a solitary lesion; post-SRS T1CE reveals enlargement; rCBV = 3.1; PSR = 80%; high risk of tumour progression based on nomogram prediction; Post-surgery T1CE shows completed resection of the right frontal lesion; Histology shows islands of viable metastatic papillary carcinoma on H&E staining at × 40 magnification. **B** Right temporal lobe metastasis lesion showing radiation necrosis: pre-SRS T1CE shows a solitary lesion; post-SRS T1CE reveals enlargement; rCBV = 0.66; PSR = 152%; low risk of tumour progression based on nomogram prediction; Post-surgery T1CE shows completed resection of the right frontal lesion; Histology shows pale areas of patchy, non-palisading necrosis with sharp demarcation from oedematous brain tissue, consistent with radiation necrosis on H&E staining at × 40 magnification

It is worth noting that in two patients, accurate assessment of rCBV and PSR was not feasible due to haemorrhagic signal susceptibility (Supplementary Fig. 2). This highlights a limitation of DSC-derived haemodynamic parameters in the presence of intertumoural bleeding. While both lesions ultimately progressed (as patients died within 1 year), whether alternative imaging techniques could offer reliable diagnostic value in such settings remains an open question. In another patient, two lesions showed more than 20% volume enlargement, both of which were included for analysis, reflecting real-world complexity in the treatment response assessment. However, rCBV and PSR calculations correctly identified the recurrence of tumour in one lesion and radiation necrosis in another lesion (Fig. 6).

Importantly, accurate differentiation between tumour recurrence and radiation-related changes is essential for optimising patient management. Misclassification may lead to unnecessary local therapies, premature discontinuation of effective systemic treatment, or erroneous attribution of progression in clinical trials investigating new therapeutic agents. T2*-weighted DSC perfusion MRI remains a valuable tool in the post-treatment assessment of brain metastases. The combined use of rCBV and PSR improves diagnostic sensitivity and specificity, supports clinical decision-making, and may help avoid overtreatment or under-treatment, ultimately improving patient outcomes.

Some limitations of this study should be acknowledged. First, the retrospective design inherently carries a risk of selection bias. Second, this was a single-centre study, which may limit the generalisability of the findings to broader clinical settings. Third, only the region of the highest mean rCBV was selected for lesion assessment to improve sensitivity in detecting focal tumour recurrence, which could be underestimated by global mean or median values derived from the entire tumour volume. Fourth, rCBV was derived from uncorrected DSC data without leakage correction, which may lead to a slight underestimation of absolute values. In this study, as many of these patients did not have longer-term radiological follow-up (12 months or greater post-SRS), definitive classification of “pseudo-progression” was not appropriate in this study. For this reason, the term “radiation necrosis” has been used in a broader sense to capture all non-progressing lesions as the presumed driver of treatment response. Future prospective multicentre studies with larger cohorts are necessary to validate these results and refine the proposed cut-off thresholds.

## Conclusion

The combined use of DSC-derived PSR and rCBV provides a practical imaging strategy for distinguishing tumour progression from radiation necrosis after SRS treatment in brain metastases. PSR, in particular, may offer added value without additional scan time or complexity. Routine incorporation of this dual-parameter model into follow-up imaging protocols may be recommended to reduce time-to-diagnosis and the need for more resource-intensive imaging techniques.

## Supplementary information

ELECTRONIC SUPPLEMENTARY MATERIAL

## Data Availability

The datasets generated and/or analysed during the current study are not publicly available due to patient confidentiality, institutional restrictions, but are available from the corresponding author on reasonable request.

## Abbreviations

AUC: Area under the curve
CIs: Confidence intervals
DCE: Dynamic contrast-enhanced
DSC: Dynamic susceptibility contrast
ICC: Intraclass correlation coefficient
MRS: Magnetic resonance spectroscopy
OR: Odds ratio
PSR: Percentage signal recovery
PET: Positron emission tomography
ROC: Receiver operating characteristic
rCBV: Relative cerebral blood volume
SRS: Stereotactic radiosurgery
TRAM: Treatment response assessment map
