# Supplementary material for: Percentage signal recovery plus relative cerebral blood volume: a practical dual-parameter strategy for differentiating post-stereotactic radiosurgery tumour progression from radiation necrosis in brain metastases
Source: Insights Imaging. 2026 May 21;17:136. doi: 10.1186/s13244-026-02240-5 (PMC13194838; doi:10.1186/s13244-026-02240-5)
Supplement: Supplementary file 1 — ELECTRONIC SUPPLEMENTARY MATERIAL [file 13244_2026_2240_MOESM1_ESM.pdf]

Percentage Signal Recovery Plus Relative Cerebral Blood Volume: A Practical Dual-Parameter Strategy for Differentiating Post-Stereotactic Radiosurgery Tumour Progression from Radiation Necrosis in Brain Metastases

ELECTRONIC SUPPLEMENTARY MATERIAL

Supplementary Table 1. Baseline clinical and imaging characteristics between the training and validation sets

	Training Set (n=38)	Validation Set (n=24)	*p* value
**Age in years (mean ± SD)**	59 ± 11	63 ± 8	0.15[a]
**Sex, male:female**	8:30	8:16	0.43[b]
**Primary tumour (count)**			0.25[b]
**Breast**	21	7	
**lung**	5	7	
**melanoma**	7	4	
**gynaecology**	1	2	
**Neuroendocrine**	1	0	
**rectal**	1	2	
**renal**	2	2	
**Nonbrain metastasis, yes:no**	6:32	2:22	0.46[b]
**Surgery on brain metastasis during follow-up, yes:no**	13:25	16:8	0.02[c*]
**Chemotherapy, yes:no**	22:16	15:9	0.92[c]
**Targeted therapy or immunotherapy, yes:no**	27:11	11:13	0.08[c]

**Follow-up, alive:dead**	16:22	13:11	0.50[c]
**rCBV (mean ± SD)**	1.68 ± 1.33	1.55 ± 1.34	0.76[d]
**PSR (mean ± SD)**	143% ± 58%	155% ± 134%	0.75[d]

[a] t test

[b] Fisher's exact test

[c] Chi-squared test

[d] Wilcoxon rank-sum test

[*] p value < 0.05

Supplementary Figure 1. Representative case demonstrating nomogram-based risk estimation.

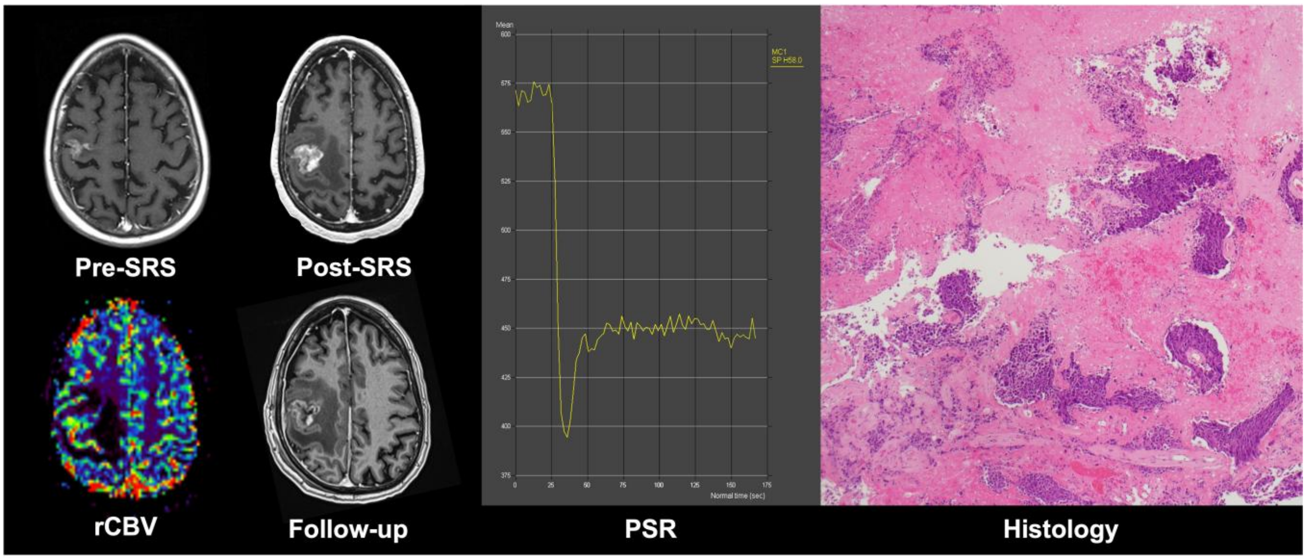

Patient_38: A 57-year-old female with a history of breast cancer who was deceased at follow-up. Pre-SRS contrast-enhanced T1-weighted image shows a metastasis in the right frontal lobe. Post-SRS T1CE at 6 months demonstrates lesion enlargement. Post-SRS T1CE at 6 months rCBV map shows a near borderline rCBV value of 1.59 and DSC-derived signal intensity-time curve demonstrates a low PSR of 34%. Follow-up post-surgical T1CE shows a persistent enhancing lesion and histology has confirmed viable perivascular tumour islands, surrounded by confluent necrosis on H&E staining at × 40 magnification. The borderline rCBV alone is inconclusive, but the markedly reduced PSR and high nomogram-predicted risk (Figure 2) support the diagnosis of tumour progression. This case highlights the complementary role of PSR in improving diagnostic confidence when rCBV is equivocal.

Supplementary Figure 2. Example of a lesion with susceptibility-related artifacts limiting perfusion analysis.

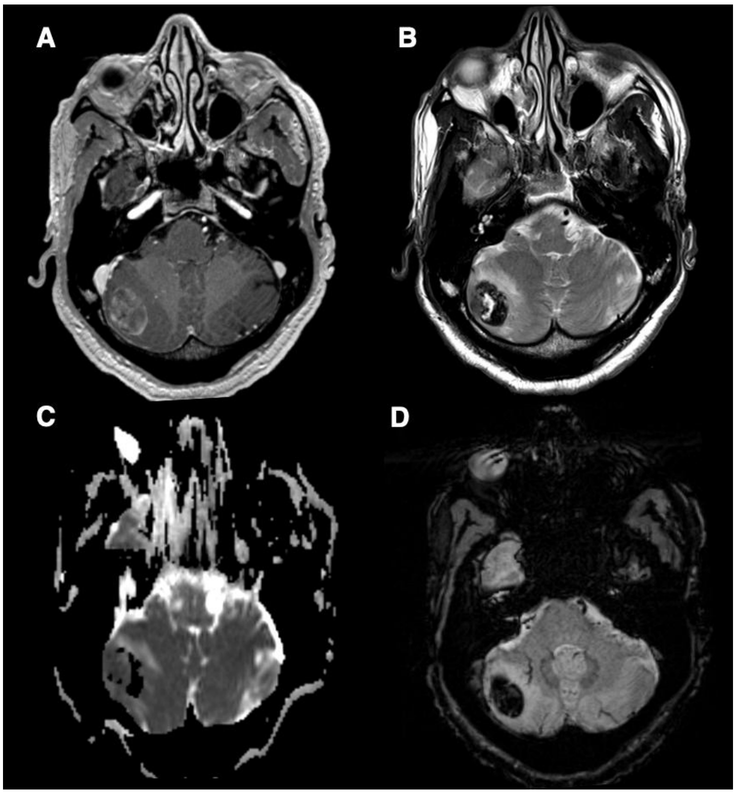

(A) Contrast-enhanced T1-weighted image showing an enhancing lesion in the right cerebellum; (B) T2-weighted image demonstrating surrounding oedema; (C) ADC map showing mildly restricted diffusion; (D) SWI revealing prominent intralesional blooming, consistent with haemorrhage. Due to susceptibility artifacts from haemorrhage, accurate assessment of rCBV and PSR was not feasible in this case, highlighting a known limitation of DSC-derived perfusion metrics in the presence of intertumoral bleeding.
